# TAAR1 agonists improve glycemic control, reduce body weight and modulate neurocircuits governing energy balance and feeding

**DOI:** 10.1016/j.molmet.2024.101883

**Published:** 2024-01-16

**Authors:** Nina Dedic, Lien Wang, Eva Hajos-Korcsok, Jacob Hecksher-Sørensen, Urmas Roostalu, Steven P. Vickers, Serena Wu, Christoph Anacker, Colleen Synan, Philip G. Jones, Snezana Milanovic, Seth C. Hopkins, Linda J. Bristow, Kenneth S. Koblan

**Affiliations:** 1 Sumitomo Pharma America, Inc., Marlborough, MA, USA; 2 Gubra ApS, Hørsholm, Denmark; 3 Sygnature Discovery, Nottingham, UK; 4 Department of Psychiatry, New York State Psychiatric Institute (NYSPI), Columbia University, NY, New York City, USA

**Keywords:** Trace amine-associated receptor 1 (TAAR1), Obesity, Antipsychotics, c-fos imaging, Homeostatic and hedonic feeding circuits

## Abstract

**Objective:**

Metabolic Syndrome, which can be induced or exacerbated by current antipsychotic drugs (APDs), is highly prevalent in schizophrenia patients. Recent preclinical and clinical evidence suggest that agonists at trace amine-associated receptor 1 (TAAR1) have potential as a new treatment option for schizophrenia. Intriguingly, preclinical tudies have also identified TAAR1 as a novel regulator of metabolic control. Here we evaluated the effects of three TAAR1 agonists, including the clinical development candidate ulotaront, on body weight, metabolic parameters and modulation of neurocircuits implicated in homeostatic and hedonic feeding.

**Methods:**

Effects of TAAR1 agonists (ulotaront, RO5166017 and/or RO5263397) on body weight, food intake and/or metabolic parameters were investigated in rats fed a high-fat diet (HFD) and in a mouse model of diet-induced obesity (DIO). Body weight effects were also determined in a rat and mouse model of olanzapine-, and corticosterone-induced body weight gain, respectively. Glucose tolerance was assessed in lean and diabetic *db/db* mice and fasting plasma glucose and insulin examined in DIO mice. Effects on gastric emptying were evaluated in lean mice and rats. Drug-induced neurocircuit modulation was evaluated in mice using whole-brain imaging of c-fos protein expression.

**Results:**

TAAR1 agonists improved oral glucose tolerance by inhibiting gastric emptying. Sub-chronic administration of ulotaront in rats fed a HFD produced a dose-dependent reduction in body weight, food intake and liver triglycerides compared to vehicle controls. In addition, a more rapid reversal of olanzapine-induced weight gain and food intake was observed in HFD rats switched to ulotaront or RO5263397 treatment compared to those switched to vehicle. Chronic ulotaront administration also reduced body weight and improved glycemic control in DIO mice, and normalized corticosterone-induced body weight gain in mice. TAAR1 activation increased neuronal activity in discrete homeostatic and hedonic feeding centers located in the dorsal vagal complex and hypothalamus with concurrent activation of several limbic structures.

**Conclusion:**

The current data demonstrate that TAAR1 agonists, as a class, not only lack APD-induced metabolic liabilities but can reduce body weight and improve glycemic control in rodent models. The underlying mechanisms likely include TAAR1-mediated peripheral effects on glucose homeostasis and gastric emptying as well as central regulation of energy balance and food intake.

## Highlights

• TAAR1 agonists, including schizophrenia drug candidate ulotaront, exhibit weight-lowering and glucoregulatory effects in rodents.
• TAAR1 activation modulates neuronal activity in discreet homeostatic and hedonic feeding centers.
• The beneficial effects of TAAR1 agonists in rodents support an improved metabolic-risk profile vs established antipsychotics.

## 1 Introduction

Over the past 15 years trace amine-associated receptor 1 (TAAR1) has attracted considerable interest as a target for neuropsychiatric disorders [[1], [2], [3], [4]]. The development of selective small molecule agonists has further reinforced the therapeutic potential of this G-protein-coupled receptor (GPCR), most prominently for the treatment of schizophrenia. TAAR1 agonists have shown robust antipsychotic-like effects in a wide range of animal models, with two drug-candidates (ulotaront and ralmitaront) advancing into Phase 2/3 clinical trials [1,4,5]. Promising efficacy and safety results in patients with schizophrenia were reported for ulotaront, a TAAR1 agonist with additional 5-HT_1A_ agonist activity, currently in Phase 3 clinical development [1,[5], [6], [7], [8], [9]]. Although the underlying mechanisms are not fully understood, preclinical evidence suggests that TAAR1-mediated modulation of monoaminergic neurotransmission, particularly presynaptic dopaminergic tone, is associated with the beneficial, centrally-mediated effects of agonist molecules [1,2,4].

TAAR1, which was discovered in 2001 [10,11], is a member of the rhodopsin-like, trace amine receptor family and activated by several endogenous trace amines including p-tyramine, β-phenylethylamine, octopamine and tryptamine [2]. TAAR1 is broadly expressed throughout the brain, although at low levels [10,[12], [13], [14], [15]]. In the periphery, its expression has been reported in the stomach, the duodenum and pancreas, including β cells [13,16,17]. Supported by its central and peripheral expression pattern, TAAR1 has recently been implicated in the regulation of metabolic function and food reward behavior [[16], [17], [18], [19]]. Selective TAAR1 agonists have been shown to decrease body weight in lean rodents, prevent olanzapine-induced weight gain in rats, reduce food intake and excess body weight in a mouse model of diet-induced obesity (DIO) and attenuate binge-like eating in rats [16,17,20,21]. Additional work in DIO and *db/db* mice (a mouse model of type 2 diabetes) also reported improved glucose tolerance and insulin sensitivity, as well as reduced plasma and liver triglyceride levels [16]. Genetic analysis in patients with psychiatric and metabolic disorders has identified several rare variants in TAAR genes, including TAAR1 [18]. Some of the variants show altered receptor function *in vitro* [22], warranting further assessment of naturally occurring TAAR1 variants in humans. The described genetic and preclinical findings are of significant relevance for potential TAAR1 agonist therapeutics since obesity, hyperglycemia, insulin resistance and dyslipidemia constitute major side effects of current antipsychotic drug (APD) classes [[23], [24], [25], [26]]. Among these, the second-generation APDs olanzapine and clozapine have the greatest propensity for inducing metabolic dysregulation although similar liabilities are also reported for some first-generation agents including haloperidol [23]. While substantial evidence has associated dopamine D_2_ and serotonin 5-HT_2A_ receptor blockade with the symptomatic benefit of APDs, the underlying targets linked to the metabolic dysregulation are far less understood and may include activity at D_2_, 5-HT_2C_, H_1_ and M_3_ receptors [27,28]. In addition, there is evidence that psychosis is associated with metabolic alterations independent of common risk factors such as antipsychotic medication and lifestyle [29,30].The need for novel treatments that lack APD class-specific metabolic side-effects is therefore apparent.

Here, we use a combination of approaches to evaluate the effects of three TAAR1 agonists on metabolic parameters in rodent models of diabetes, obesity, and iatrogenic weight gain. We corroborate previous results showing beneficial effects of TAAR1 activation on glycemic control and body weight and demonstrate that this is a class effect seen across structurally distinct TAAR1 agonist compounds. Furthermore, we provide novel mechanistic insight into TAAR1-mediated metabolic regulation by showing that TAAR1 agonists modulate neural activity of homeostatic and hedonic neurocircuits governing energy balance and feeding. The current data further supports evaluation of TAAR1 agonists for the treatment of metabolic disorders including metabolic dysregulation in patients with schizophrenia.

## 2 Materials and methods

### 2.1 Animals

Mice used for the gastric emptying (acetaminophen absorption test) and c-fos imaging studies were treated in accordance with the National Danish legislation BEK 2028 of 14/12/2020, which is based on the Directive 2010/63/EU of the European Parliament and of the Council of 22 September 2010 on the protection of animals used for scientific purposes. Evaluation of compound effects in the chronic corticosterone model was approved by the Institutional Animal Care and Use Committee (IACUC) at the New York State Psychiatric Institute (NYSPI). For all other studies, animal procedures were performed in accordance with UK regulations, as detailed in the Animals (Scientific Procedures) Act 1986. Unless otherwise specified, animals were housed under a 12-h:12-h light/dark cycle in a temperature and humidity-controlled environment and with access to standard chow diet and tap water *ad libitum*.

### 2.2 Test articles

Ulotaront (SEP-363856), RO5166017 and RO5263397 were synthesized at Sumitomo Pharma America. Glucagon-like peptide 1 receptor (GLP-1R) agonists exendin-4 and liraglutide were purchased from Tocris and semaglutide from Nomeco. Acetaminophen, phenol red and corticosterone were obtained from Sigma-Aldrich and the atypical antipsychotic olanzapine from Glentham Life Science. Ulotaront was dissolved in sterile water or saline for oral and intraperitoneal administration, respectively. Ulotaront doses are expressed as the free base and were corrected for the salt content. RO5166017 and RO5263397 (both free base) were dissolved in 0.3%Tween80 in sterile water or in 0.3% Tween 80 in saline for oral and intraperitoneal administration, respectively. Dosing preparations for all other test articles are specified throughout the text.

### 2.3 Glucose tolerance tests

The oral glucose tolerance test (oGTT) was conducted in lean, male C57BL/6J (10 weeks; Charles River Laboratories (CRL), Margate, UK) and male *db/db* mice (6–7 weeks; CRL, Calco, Italy). Lean, male CD1 mice (10 weeks; CRL, Margate, UK) were used for the intravenous (iv) GTT. Mice were singly housed and fasted 16 h prior to the glucose tolerance test. During the oGTT, mice were dosed with ulotaront (0.3, 1, 3 or 10 mg/kg, po) or vehicle 30 min prior to oral glucose administration (2.0 g/kg). Exendin-4 (dissolved in 1× phosphate buffered saline (PBS)) was included as a positive control and administered to lean C57BL/6J (1 μg/kg, ip) or *db/db* (300 μg/kg, ip) mice 10 min prior to the glucose load. Blood samples were collected at baseline (prior to test article dosing), 3 min before glucose administration, and at 15, 30, 60 and 120 min post-glucose administration. During the ivGTT, mice were dosed with ulotaront (0.3, 1, 3 or 10 mg/kg, po) or vehicle 30 min prior to intravenous glucose administration (1 g/kg). Exendin-4 (40 μg/kg dissolved in 1× PBS) was dosed iv concomitantly with the glucose. Blood samples were collected at baseline, 3 min before glucose administration, and at 5, 10, 30 and 60 min post-glucose administration. Plasma was separated by centrifugation and subsequently analyzed for glucose (glucose hexokinase reagent, Thermo Fisher) and insulin (insulin ELISA, Alpco) levels.

### 2.4 Assessment of gastric emptying

#### 2.4.1 Acetaminophen absorption test (ATT)

Lean, male C57BL/6JRj mice (8–9 weeks, JanVier, Le Genest-Saint-Isle, France) were semi-fasted (∼50% of prior food intake) over-night and dosed orally with ulotaront (0.3, 1, 3 or 10 mg/kg), RO5166017 (0.1, 0.3, 1 mg/kg), RO5263397 (0.01, 0.1, 1 mg/kg) or the corresponding vehicle 30 min prior to acetaminophen administration (160 mg/kg, po suspended in 1.5% (w/v) HPMC/1.5% (w/v) HPCD in water). Semaglutide (10 nmol/kg, s.c. dissolved in 1× PBS with 0.1% BSA) was included as a positive control and administered 30 min prior to acetaminophen. Blood samples were collected at baseline (prior to test article dosing) and at 10, 30, 60 and 120 min post-acetaminophen administration. Serum was separated by centrifugation and acetaminophen concentrations determined using a commercially available kit (TDM Acetaminophen Gen.2, Roche Diagnostics) followed by quantification in the Cobas c 501 autoanalyzer. The ACET2 calibrator (Roche Diagnostics) was used to generate the standard curve.

#### 2.4.2 Phenol red test

Lean, male C57BL/6J mice (8–9 weeks; CRL, Margate, UK) or Sprague Dawley rats (200–250 g CRL, Margate, UK) were food and water were restricted ∼22 and 2 h prior to phenol red administration, respectively. Animals were dosed orally with ulotaront (0.3, 1, 3 or 10 mg/kg), RO5166017 (mice, 0.3 mg/kg; rats, 0.3, 1, 3 and 10 mg/kg), RO5263397 (mice, 0.1 mg/kg; rats, 1, 3, 10 and 30 mg/kg) or the corresponding vehicle 30 min prior to phenol red (0.05% w/v in 1.5% aqueous HPMC, po) administration. Thirty minutes later, animals were terminated, and the remaining phenol red recovered from the stomach. The amount of phenol red was determined from the absorbance of the sample assessed at 560 nm by spectrophotometer (SpectraMax iD5 Molecular Devices) against distilled water. A separate group of vehicle-treated animals (of the same strain and age or weight), terminated immediately after dosing with phenol red, served as standard controls (i.e., maximal 100% absorbance). The percent gastric emptying 30 min post-phenol red dosing for each mouse was calculated as: [1 − (individual absorbance of the sample/mean absorbance of standard controls)] × 100.

### 2.5 Studies in rats fed a high-fat diet (HFD)

Three separate studies were conducted in female Sprague Dawley (SD) rats obtained from CRL (Margate, UK). Free access to HFD (VRF1 plus 20% lard; Special Diet Service) was initiated two weeks prior to test article administration. Rats were maintained on a 8-h:16-h reversed light–dark cycle.

In the first study, rats (200–300 g at the start of HFD exposure) were dosed once daily with ulotaront (1, 3 or 10 mg/kg, po) or vehicle for 14 consecutive days. Olanzapine (3 mg/kg, po suspended in 1% methyl cellulose; qd × 14 days) was included as a positive control. Body weights, food and water intake were measured daily. On day 15, following a 16 h fast, liver tissue was collected 4 h after test article administration for analysis of liver triglycerides using a Cobas c111 analyzer (Roche Diagnostics). Carcass composition (i.e., fat content) of each animal was determined using the FoodScan NIR (near infra-red) meat analyzer (Foss). Frozen rat carcasses were placed in liquid nitrogen and then individually milled using a Retsch SM2000 Laboratory cutting mill (Cristison Scientific Equipment Ltd) precooled using solid carbon dioxide. Subsequently, a portion of the milled sample (∼50 g) was placed in the FoodScan analyser and scanned using the default settings.

In the second study, female SD rats (185–245 g at the start of HFD exposure) were initially dosed once daily with olanzapine (3 mg/kg, po) or vehicle for 7 consecutive days. On day 8, olanzapine-treated rats were switched to ulotaront (0.3, 1 or 3 mg/kg, po, qd) or vehicle treatment for an additional 7 days while one group continued to receive olanzapine. Rats dosed with vehicle during the first 7 days continued to receive vehicle treatment. Body weights, food and water intake were measured daily. On day 15, following a 16 h fast, animals were terminated, and carcasses frozen for subsequent carcass composition analyses.

RO5263397 (1 or 10 mg/kg, po, qd) was evaluated in female SD rats (200–300 g at the start of HFD exposure) under the same testing conditions as in study two, except that body fat analysis was not conducted.

### 2.6 Evaluation in chronic corticosterone-treated mice

Male C57BL/6J mice (8 weeks, Jax, Bar Harbor, MA, USA) were initially administered corticosterone (35 μg/ml) or vehicle (0.45% β-cyclodextrin) *ad libitum* in drinking water for 28 days. Subsequently, corticosterone-treated mice were orally dosed with ulotaront (1, 5 or 10 mg/kg) or vehicle, once daily for 21 days. Animals continued to receive corticosterone in the drinking water throughout the drug treatment period. Body weights were determined weekly.

### 2.7 Evaluation in DIO mice

Obesity was induced in male, C57BL/6J mice (6–7 weeks; CRL, Margate, UK) by providing animals with *ad libitum* access to a high fat diet (D12451; 20% protein, 35% carbohydrate and 45% fat; Research Diets) starting 16 weeks prior to study start. Animals were maintained on a 8-h:16-h reversed light–dark cycle. Mice were dosed once daily with ulotaront (0.3, 1, 3 or 10 mg/kg, po) or vehicle for 35 consecutive days. Liraglutide (0.2 mg/kg, s.c., qd), was included as a positive control. Body weight, food and water intake were assessed daily. Fasting plasma glucose and insulin were measured on day 15 following a 4 h fast. Plasma glucose levels were determined with the Infinity Glucose Hexokinase reagent (Thermo Scientific). Insulin was measured using a mouse insulin ELISA (Alpco). HOMA-IR was calculated as follows: HOMA-IR = fasting glucose (mM) × fasting insulin (μU/ml)/22.5.

### 2.8 Whole brain c-fos imaging

Male C57BL/6JRj mice (8 weeks; JanVier, Le Genest-Saint-Isle, France) received a single administration of ulotaront (3 mg/kg, ip), RO5263397 (0.3 mg/kg, ip) or the respective vehicle 105 min prior to the transcranial perfusion (heparinized PBS and 10% neutral buffered formalin (NBF)). Subsequently, the brains were dissected and postfixed overnight in 10% NBF at room temperature. The iDISCO+ (immunolabeling-enabled three-dimensional imaging of solvent-cleared organs) [31,32] protocol was used for whole brain immunolabelling as previously described [33,34]. For visualization of c-fos expression, rabbit anti-c-fos antibody (1:5000, Cell Signaling Technology) was used followed by incubation with the secondary donkey anti rabbit Cy-5 antibody (1:1000, Jackson ImmunoResearch). The optically transparent brain samples were imaged using LaVision ultramicroscope II (Miltenyi Biotec). Image processing, registration and cell detection was performed according to the method of Perens and colleagues [33]. In addition to voxel-level analysis, a total of 839 brain regions of interest (ROIs) were analyzed.

### 2.9 Statistical analysis

Statistical analysis was performed using Graphpad Prism v8.0. Data are presented as means ± SEM or + SEM. p ≤ 0.05 was considered statistically significant. Time-course data were analyzed by mixed-effects model or two-way repeated measures ANOVA followed by appropriate post-hoc comparisons (i.e., Dunnett's or Tukey's post hoc test). Individual group comparisons were performed with an unpaired, two-tailed t-test. Multiple group comparisons were conducted with one-way ANOVA followed by Dunnett's or Tukey's post-hoc tests, as appropriate. ROI-based and voxel-wise statistical analysis were performed to compare the effect of treatment versus vehicle on c-fos expression as previously described [33,34]. For the ROI analysis, a Negative Binomial Generalized Linear Model (GLM) was fitted to the cell count data, and a Dunnett's test was performed for multiple group comparisons within each region. Voxel-wise statistics are provided as Z-scores based on c-fos-positive density maps. The density maps were generated for each sample by calculating the average number of c-fos-positive cells within a radius of 100 μm at each voxel position. Hereafter, Welch's t-test was applied, at each voxel-position, and the resulting p-value was converted to a Z-score.

## 3 Results

### 3.1 TAAR1 agonist ulotaront improves glucose tolerance

**Figure 1:**
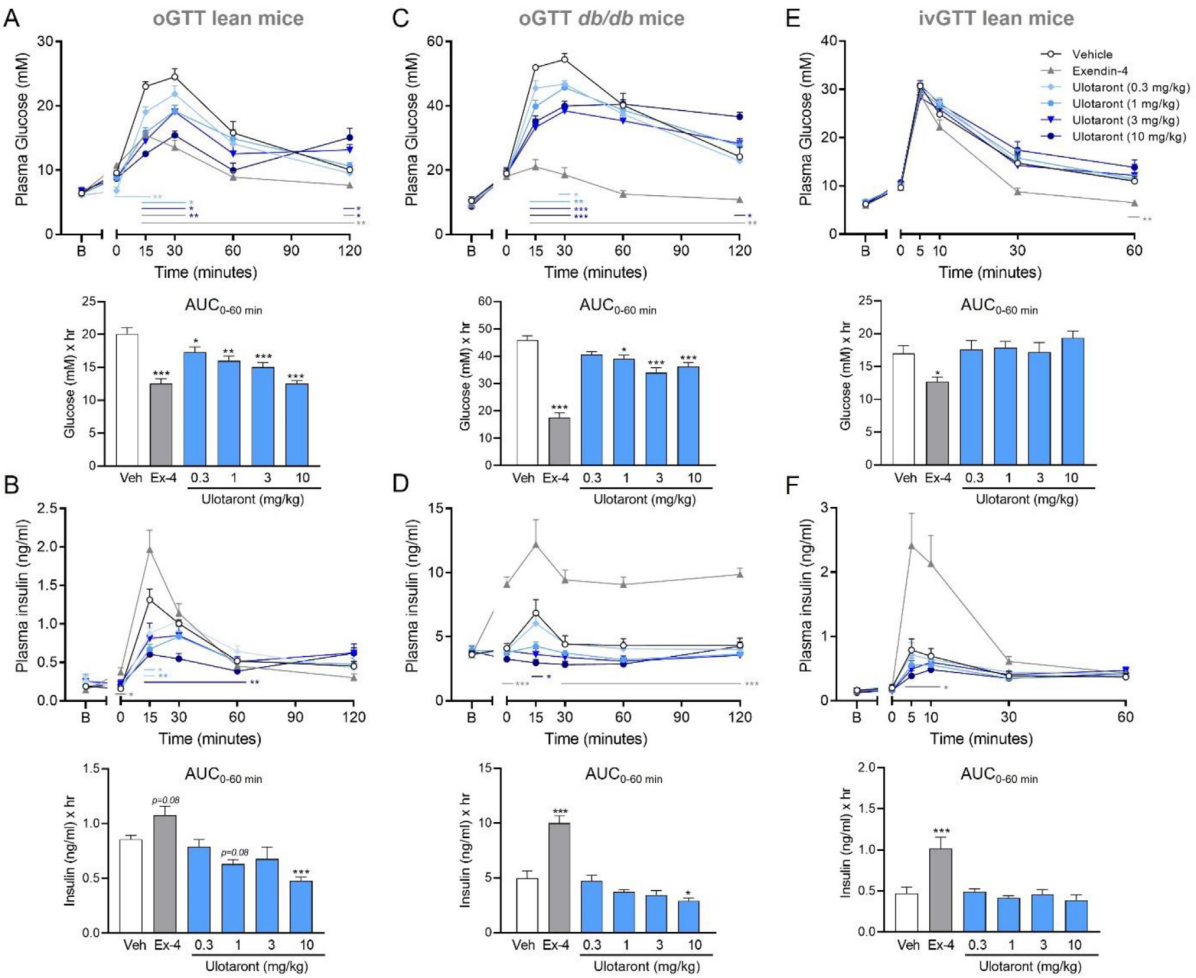
**Ulotaront improves oral glucose tolerance in lean and *db/db* mice.** (A) Single oral administration of ulotaront significantly and dose-dependently decreased glucose (A) and insulin (B) excursion during the first 60 min (AUC_0–60 min_) of the oGTT in lean male mice. Similar effects on glucose (C) and insulin (D) time course and AUC_0–60 min_ were also seen in *db/db* mice. No significant effect on glucose (E) and insulin (F) levels were noted during an ivGTT in lean mice. B = baseline, prior to compound dosing. Glucose (2 g/kg, po or 1 g/kg, iv) was administered immediately after sample collection at timepoint 0. Ex-4 = exendin-4 (1 μg/kg, ip (A–B), 300 μg/kg, ip (C–D) or 40 μg/kg, iv (E–F)). Data are means + SEM. Mixed-effects analysis (time-course data) or one-way ANOVA (AUC data) followed by Dunnett's multiple comparisons test; ∗p < 0.05, ∗∗p < 0.01, ∗∗∗p < 0.001 vs vehicle (Veh); N = 9–10/group.

**Supplementary Figure 1:**
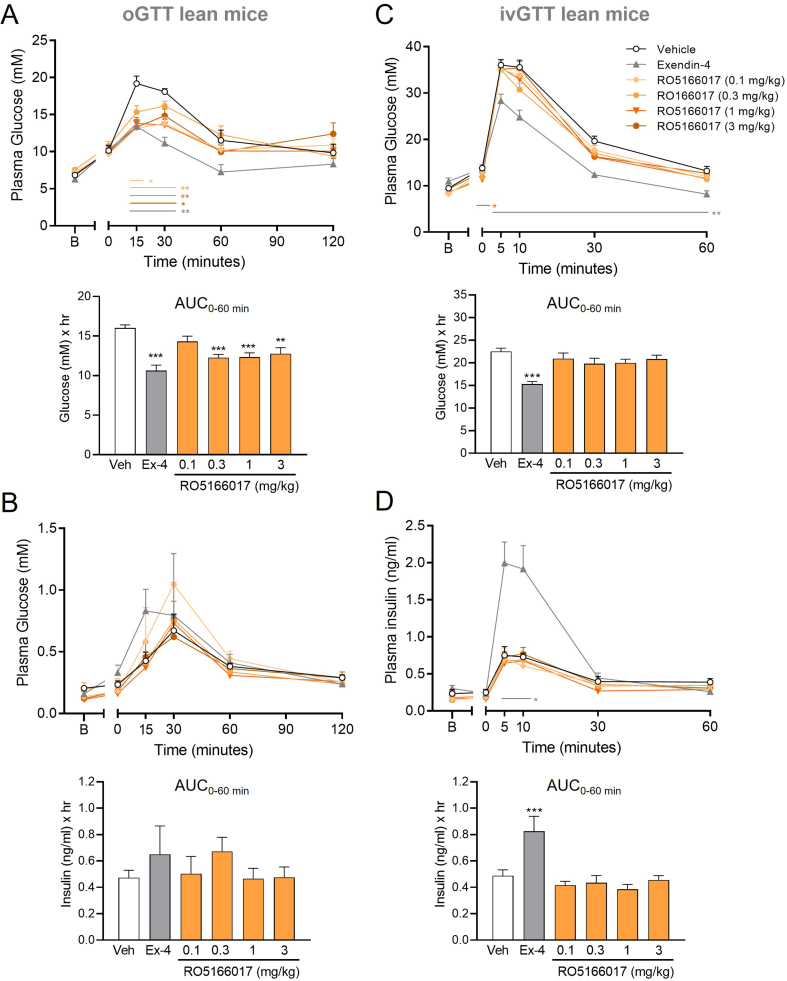
RO5166017 improves oral glucose tolerance in lean mice. (A) Single oral administration of RO5166017 in male mice significantly and dose-dependently decreased glucose (A) excursion during the first 60 min (AUC_0–60 min_) of the oGTT, with no effects seen on insulin levels (B). No significant effect on glucose (C) or insulin (D) levels were noted for RO5166017 during an ivGTT in lean mice. B = baseline, prior to compound dosing. Glucose (2 g/kg, po or 1 g/kg, iv) was administered immediately after sample collection at timepoint 0. Ex-4 = exendin-4 (1 μg/kg, ip) (A–B); 40 μg/kg, iv (C–D). Data are means + SEM. Mixed-effects analysis (time-course data) or one-way ANOVA (AUC data) followed by Dunnett's multiple comparisons test; ∗p < 0.05, ∗∗p < 0.01, ∗∗∗p < 0.001 vs vehicle (Veh); N = 9–10/group.

To assess the effects of acute ulotaront administration on glycemic control, we initially conducted an oral glucose tolerance test (oGTT) in lean C57BL/6J and *db/db* mice. *Db/db* mice, which are considered a model of type 2 diabetes, are homozygous for the spontaneous *db* mutation in the leptin receptor gene (*Leprdb*) and develop chronic hyperglycemia, insulin resistance and obesity [35]. In both C57BL/6J and *db/db* mice, single oral administration of ulotaront (0.3, 1, 3 or 10 mg/kg) dose-dependently and significantly reduced plasma glucose and insulin excursion (Figure 1A–D). The effect was most prominent during the initial 60 min post-glucose administration, as shown by the area under the curve (AUC_0__–__60 min_). At the end of the test (120 min post glucose administration), plasma glucose was significantly elevated compared to vehicle treated controls, particularly in mice that received the highest doses of ulotaront (3 and 10 mg/kg po). This type of oGTT profile suggests that the glucose-lowering effect of acute ulotaront treatment is insulin-independent and may be mediated through effects on gastric emptying. To further examine this, we conducted an ivGTT in lean CD1 mice. Intravenous administration of glucose controls for compound effects on gastric emptying and incretin hormones that are released upon oral glucose administration. In this case, single oral administration of ulotaront (0.3, 1, 3 or 10 mg/kg) had no effect on either glucose or insulin excursion in the ivGTT (Figure 1E–F). Importantly, we observed that the selective TAAR1 agonist RO5166017 (0.1, 0.3, 1 or 3 mg/kg, po) produced very similar effects to ulotaront in both the oGTT and ivGTT assays (Supplementary Figure 1). As expected, exendin-4 significantly reduced plasma glucose compared to vehicle-treated controls across all studies. In line with the profile of GLP-1R agonists, this reduction was insulin-dependent as shown by the significant increase in plasma insulin at the 15 min (oGTT) and 5 min (ivGTT) time points across most studies (Figure 1 and Supplementary Figure 1). The only exception was the oGTT study conducted with RO5166017, where neither RO5166017 nor exendin-4 significantly altered insulin excursion, likely due to the high inter-animal variability (Supplementary Figure 1B). Overall, the results demonstrate that TAAR1 agonists improve oral glucose tolerance in mice.

### 3.2 TAAR1 agonists delay gastric emptying

**Figure 2:**
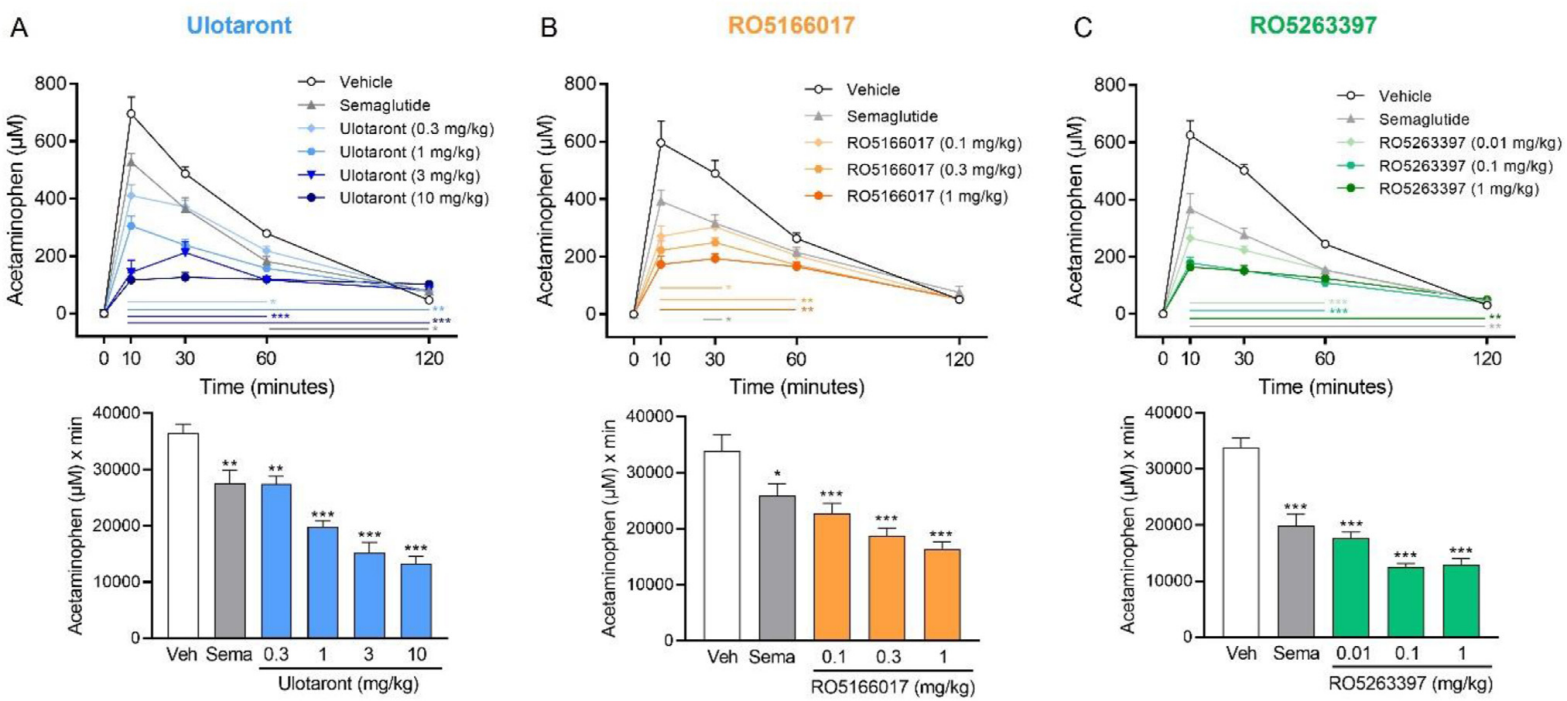
**TAAR1 agonists delay gastric emptying in mice.** Acetaminophen time-course and AUC_0–120 min_ was significantly and dose-dependently decreased following single oral administration of ulotaront (A), RO5166017 (B) or RO5263397 (C) indicating a delay in gastric emptying. Sema = semaglutide (10 nmol/kg, s.c.). Data are means + SEM. Mixed-effects analysis (time-course data) or one-way ANOVA (AUC data) followed by Dunnett's multiple comparisons test; ∗p < 0.05, ∗∗p < 0.01, ∗∗∗p < 0.001 vs vehicle (Veh); N = 7–11/group.

**Supplementary Figure 2:**
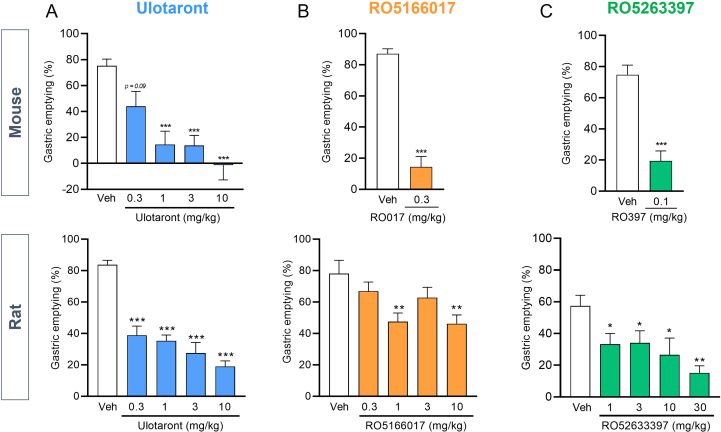
TAAR1 agonists delay gastric emptying in mice and rats. Gastric emptying, assessed with the phenol red test in mice and rats, was significantly decreased following single oral administration of ulotaront (A), RO5166017 (B) or RO5263397 (C). Data are means + SEM. One-way ANOVA followed by Dunnett's post-hoc test (for multiple groups comparisons) or unpaired t-test (single comparisons); ∗p < 0.05, ∗∗p < 0.01, ∗∗∗p < 0.001 vs vehicle (Veh); N = 8/group.

To further elucidate the mechanism contributing to the glucose-lowering effect of ulotaront in the oGTT, we examined its effect on gastric emptying in lean, C57BL/6J mice. The study was conducted using the acetaminophen absorption test, a clinically established method for the assessment of gastric emptying [36]. Single oral administration of ulotaront dose-dependently and significantly reduced serum acetaminophen concentrations indicating delayed gastric emptying at all dose levels tested (Figure 2A). Delayed gastric emptying was also observed in mice treated with the selective TAAR1 agonists RO5166017 and RO5263397 (Figure 2B–C). The tested doses of RO5166017 and RO5263397 are consisted with those reported in previous *in vivo* pharmacology studies [17,37]. In line with reported effects of GLP-1R agonists [[38], [39], [40]], semaglutide delayed gastric emptying compared to vehicle-treated controls across all studies. Lastly, we also evaluated gastric emptying using the phenol red test, a frequently utilized method in rodents that determines the recovery of phenol red directly from the stomach [41]. Consistent with prior results, oral administration of ulotaront, RO5166017 and RO5263397 significantly delayed gastric emptying in mice (Supplementary Figure 2). All three compounds also delayed gastric emptying in rats (Supplementary Figure 2). Collectively, the results demonstrate that TAAR1 agonists delay gastric emptying in rodents, supporting the hypothesis that this mechanism contributes to the glucose-lowering effects of ulotaront and RO5166017 in the mouse oGTT.

### 3.3 Ulotaront reduces body weight and food intake in rats fed a high-fat-diet

**Figure 3:**
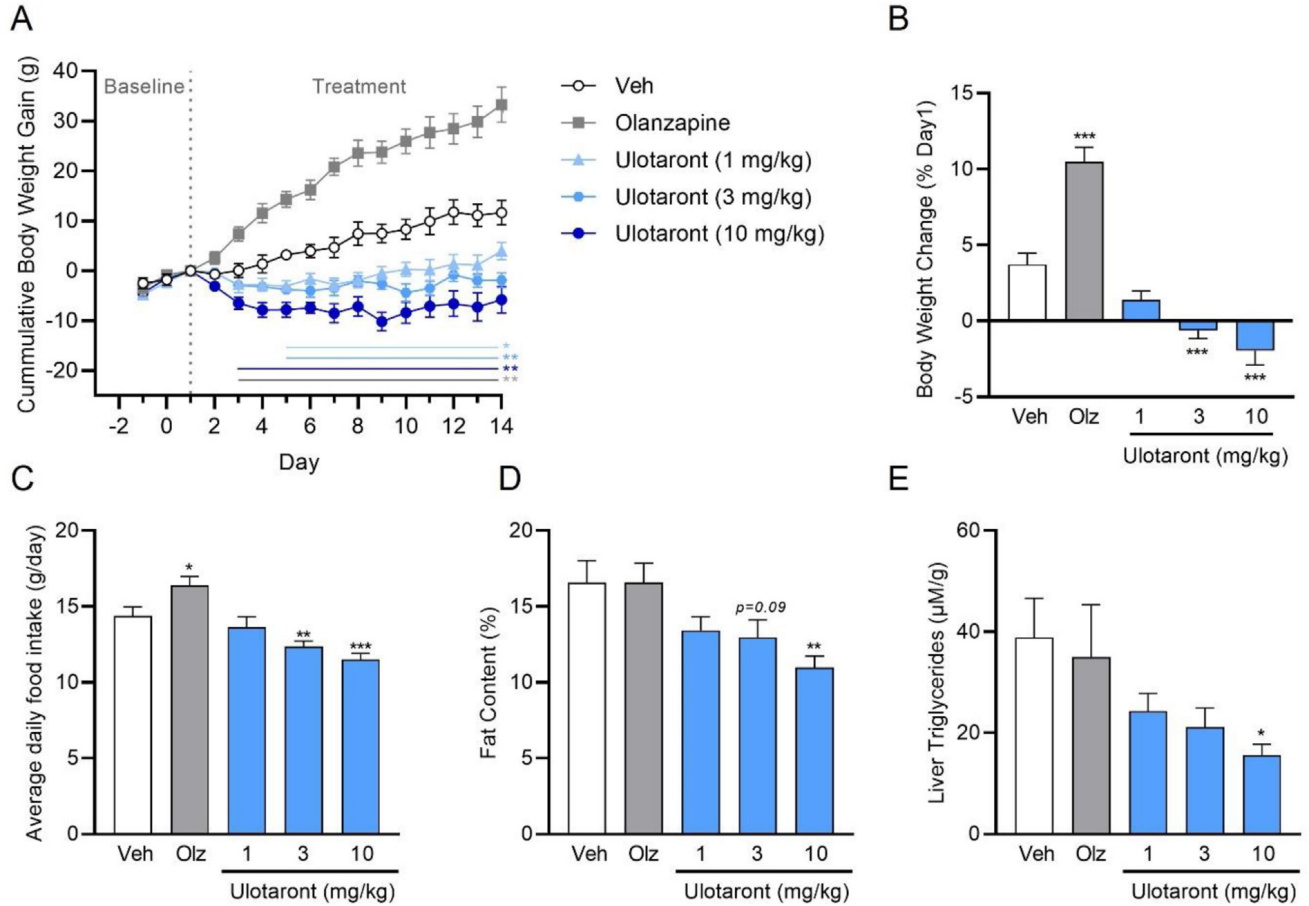
**Ulotaront reduces body weight and improves metabolic parameters in rats fed a high fat diet.** Once, daily administration of ulotaront (po) resulted in a dose-dependent reduction in (A) cumulative body weight gain, (B) percent body-weight change, (C) average daily food intake, (D) fat content and (E) liver triglyceride levels compared to vehicle controls. Olz = olanzapine (3 mg/kg, po, qd). Data are means ± or + SEM. 2-way repeated measures ANOVA (A) or one-way ANOVA (B–E) followed by Dunnett's post-hoc test; ∗p < 0.05, ∗∗p < 0.01, ∗∗∗p < 0.001 vs vehicle (Veh); N = 12/group.

**Supplementary Figure 3:**
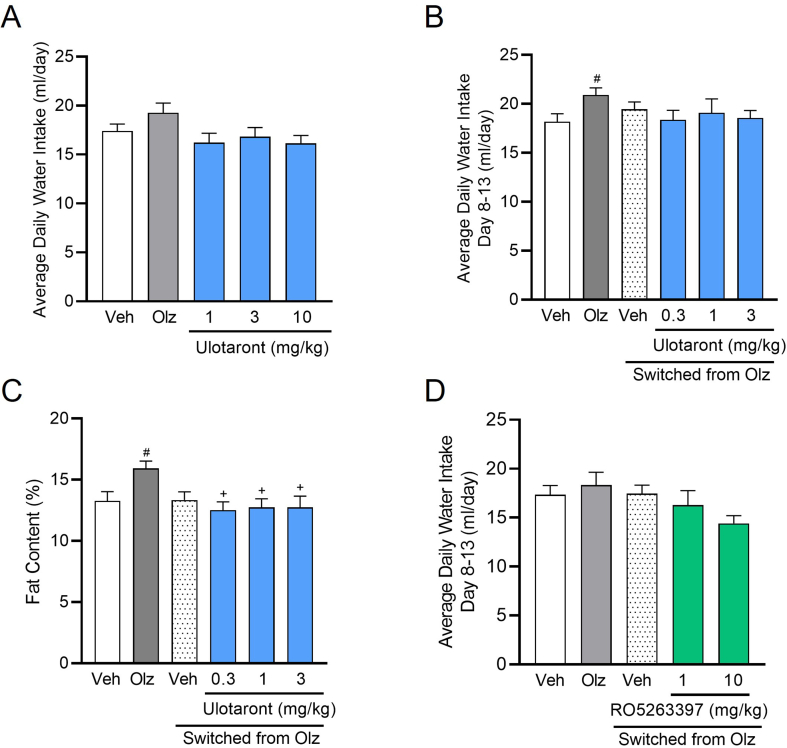
Effect of ulotaront and RO5263397 on water intake or fat content in rats fed a HFD. Average daily water intake during (A) the 14 days of ulotaront treatment (1, 3 or 10 mg/kg, po, qd) described in Figure 3 and (B) following treatment switch from olanzapine to ulotaront (0.3, 1 or 3 mg/kg, po, qd / Day 8–14) described in Figure 4. (C) Treatment switch to ulotaront significantly reduced the olanzapine-induced increase in fat content determined ex vivo at the end of the experiment. (D) Average daily water intake following treatment switch from olanzapine to RO5263397 (1 or 10 mg/kg, po, qd / Day 8–14) described in Figure 4. One-way ANOVA followed by Dunnett's (A) or Tukey's (B–D) post-hoc test; +p < 0.05 vs Olz (olanzapine); Comparisons between vehicle (D1-14) and olanzapine (D1-14) groups in B–C were analyzed separately using unpaired t-test; #p < 0.05 vs Veh (vehicle). N = 12–13.

We next investigated the effects of sub-chronic (once daily for 14 days) ulotaront treatment on body weight, food and water intake in female Sprague Dawley rats fed a HFD. The atypical antipsychotic, olanzapine, which induces prominent weight gain in rodents and humans [42], was included as a positive control. Ulotaront dose-dependently and significantly (1, 3 and 10 mg/kg/day, po) reduced cumulative body weight gain compared to vehicle treated HFD controls (Figure 3A–B). The most prominent weight decrease was observed during the initial 3–4 days of dosing after which body weight stabilized but did not increase as in vehicle-treated rats. The decrease in body weight gain was also associated with significant fat loss, determined at study end (Figure 3D), and reduced average daily food intake (Figure 3C) while water consumption was not altered (Supplementary Figure 3A). In addition, liver triglycerides measured at study end were dose-dependently and significantly reduced by ulotaront (Figure 3E). In line with previous results [43,44], treatment with olanzapine (3 mg/kg/day, po) resulted in a significant increase in body weight gain and average daily food intake. No statistically significant effects of olanzapine treatment were seen on average daily water intake (Supplementary Figure 3A), terminal fat content (Figure 3D) or liver triglyceride levels (Figure 3E) in this study.

### 3.4 Switch to ulotaront or RO5263397 reverses olanzapine-induced body weight gain and food intake in rats fed a high-fat-diet

**Figure 4:**
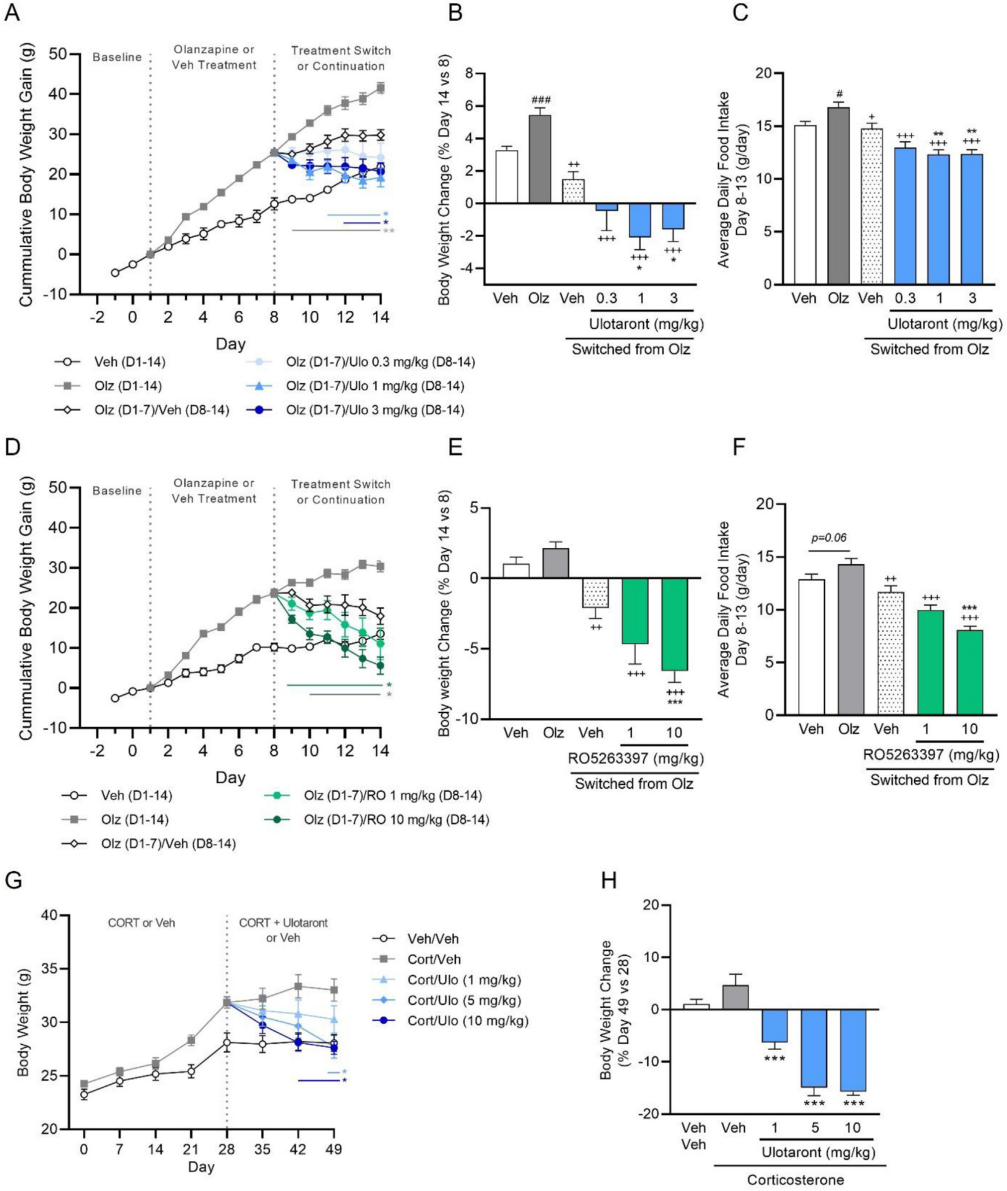
**TAAR1 agonism reverses olanzapine and corticosterone-induced body weight gain.** Once, daily olanzapine treatment (3 mg/kg, po) for 8 days in female rats maintained on a HFD significantly increased body weight gain by day 8 compared to vehicle (A,D). Rats switched from olanzapine to ulotaront treatment (0.3, 1 or 3 mg/kg, po, qd) on day 8 showed a significantly greater reduction in body weight (A, B) and average daily food intake (C) compared to rats that were switched to vehicle. In the same model, switch to RO5263397 (1 or 10 mg/kg, po, qd) also produced a greater reduction in body weight (D,E) and food intake (F) compared to a switch to vehicle. Two-way repeated measures ANOVA (A,D) or one-way ANOVA (B,C,E,F) followed by Tukey's post-hoc test; ∗p < 0.05, ∗∗p < 0.01, ∗∗∗p < 0.001 vs Olz/Veh switch; +p < 0.05, ++p < 0.01 +++p < 0.001 vs Olz. Comparisons between the vehicle (D1-14) and olanzapine (D1-14) group were analyzed separately using a two-tailed t-test; #p < 0.05, ###p < 0.001 vs Veh. N = 12–13/group. Single, daily ulotaront administration (1, 5 and 10 mg/kg, po) dose-dependently decreased body weight (G) and percent body weight gain (H) in male mice treated with chronic corticosterone (35 μg/ml in drinking water). Data are means ± or + SEM. Two-way repeated measures ANOVA (G) or one-way ANOVA (H) followed by Dunnett's post-hoc test; ∗p < 0.05, ∗∗p < 0.01, ∗∗∗p < 0.001 vs Cort/Veh; #p < 0.05 vs Veh/Veh. N = 8–12/group.

**Supplementary Figure 4:**
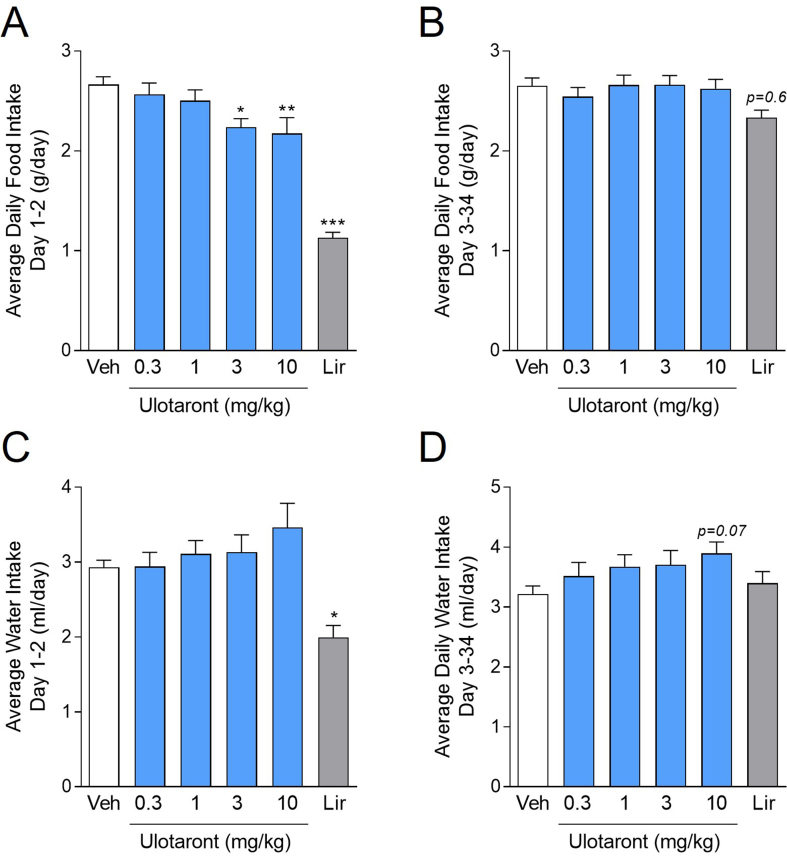
Effect of ulotaront on food and water intake in male DIO mice. Average daily food intake during (A) the first two days of treatment and (B) throughout days 3–34. Average daily water intake during (C) the first two days of treatment and (D) throughout days 3–34. Ulotaront (0.3, 1, 3 or 10 mg/kg, po, qd); Lir = Liraglutide (0.2 mg/kg, s.c., qd). Data are means + SEM. One-way repeated measures ANOVA followed by Dunnett's post-hoc test; ∗p < 0.05, ∗∗p < 0.01, ∗∗∗p < 0.001 vs vehicle (Veh); N = 12/group.

Previous studies have reported that co-administration of olanzapine with RO5263397 or ulotaront attenuates olanzapine-induced weight gain in rodents [17,21]. In order to recapitulate a more clinically relevant scenario, where switching of APDs is common, we examined whether ulotaront can reverse the metabolic effects produced by prior olanzapine treatment in female rats maintained on HFD. Except for the vehicle-treated control group, all rats were initially dosed with olanzapine (3 mg/kg, po) once daily for 7 consecutive days. On day 8, rats either continued on olanzapine treatment or were switched to treatment with vehicle or ulotaront (0.3, 1 or 3 mg/kg, po, qd) for a further 7 days. Compared to vehicle-treated controls, a significant increase in body weight gain was observed following 7 days of olanzapine treatment (Figure 4A). Rats that were switched to vehicle treatment showed a significant reduction in cumulative body weight gain (Figure 4A–B) and average daily food intake (Figure 4C) throughout the remaining study period (day 8–14) compared to animals that continued to receive olanzapine. Switching to ulotaront also significantly decreased subsequent body weight gain and food intake at all doses compared to the olanzapine-treated group. Notably, the reduction in body weight and food intake was significantly greater in rats switched to ulotaront (1 and 3 mg/kg, po) compared to those switched to vehicle treatment. In this study a significant increase in average daily water intake was observed in rats continuing olanzapine treatment on days 8–13, which was not observed in rats switched to vehicle or ulotaront (Supplementary Figure 3B). In addition, switching to ulotaront was associated with a significant reduction in fat content at all doses compared to olanzapine-treated rats (Supplementary Figure 4C). Next, we evaluated the effects of RO5263397 under similar testing conditions. Consistent with the ulotaront findings, switching from olanzapine to RO5263397 (1 or 10 mg/kg, po, qd) reduced body weight gain (Figure 4D–E) and average daily food intake (Figure 4F) and these effects were significantly greater than switching to vehicle at the highest dose.

### 3.5 Ulotaront reverses corticosterone-induced weight gain in mice

Prolonged exposure to elevated glucocorticoid levels (i.e., cortisol) caused by chronic stress, pituitary tumors or administration of steroid drugs has been associated with metabolic dysregulation including weight gain and obesity [45,46]. In rodents, chronic exposure to corticosterone (Cort) mimics some of the metabolic alterations and has been used to model stress-induced behavioral changes commonly associated with depression such as enhanced anxiety-related behavior [47,48]. Therefore, we evaluated whether ulotaront would also have an impact on Cort-induced weight gain in male C57BL/6J mice. Chronic administration of Cort (35 μg/ml) produced a significant increase in body weight that was apparent from day 21 and was relatively stable from day 28 onwards compared to vehicle controls (Figure 4G). Once, daily administration of ulotaront (1, 5 or 10 mg/kg, po), beginning on day 28, significantly and dose-dependently reduced the Cort-induced body weight gain over the treatment period (Figure 4G–H). By day 49, the body weight of mice receiving ulotaront at 5 or 10 mg/kg was similar to that seen in vehicle-treated mice that were not exposed to Cort in the drinking water (Figure 4G).

### 3.6 Ulotaront reduces body weight and improves glycemic control in DIO mice

**Figure 5:**
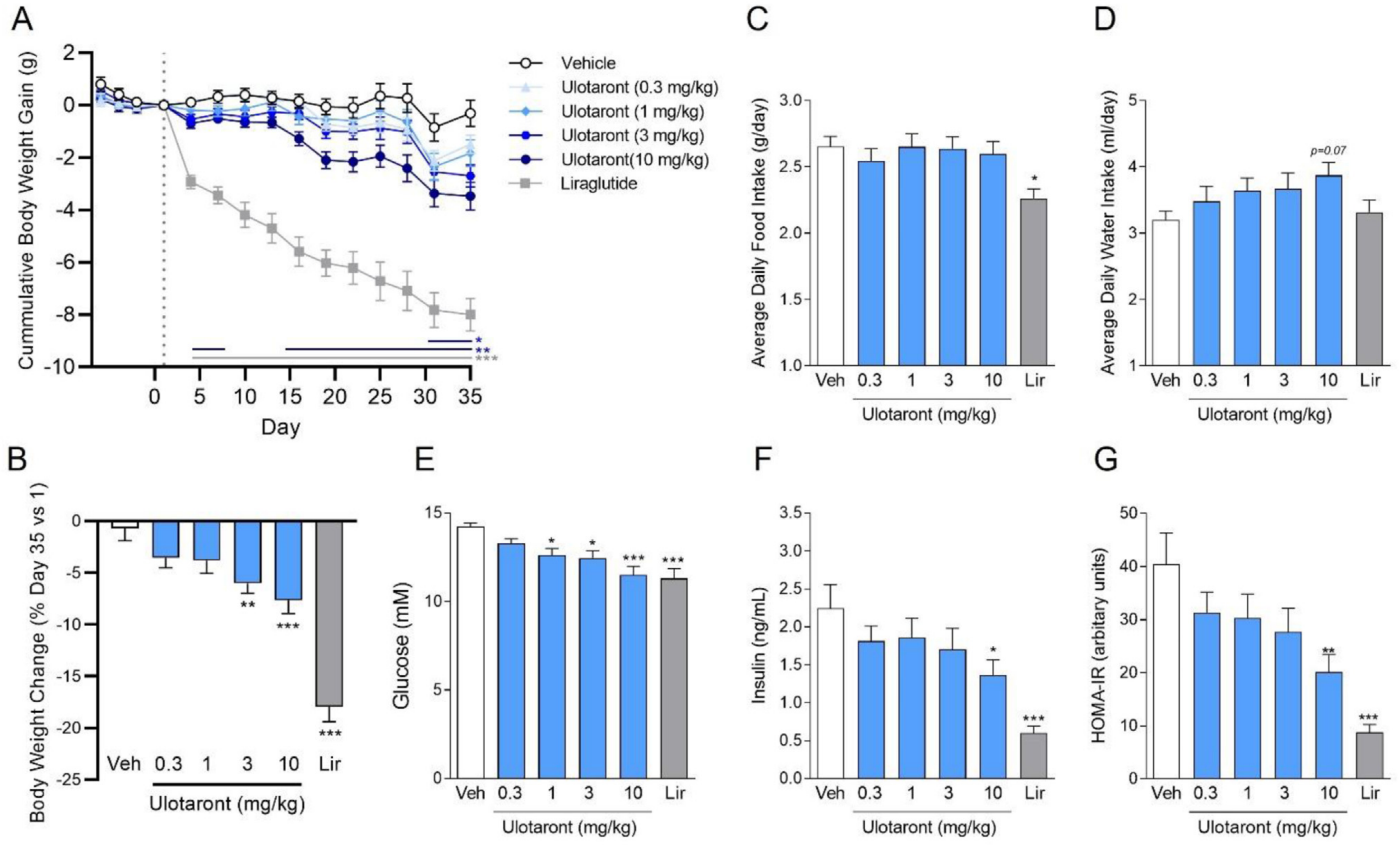
**Ulotaront reduces body weight and improves glycemic control in a mouse model of diet-induced obesity.** Once, daily ulotaront administration (0.3, 1, 3 and 10 mg/kg, po) dose-dependently reduced cumulative (A) and percent (B) body weight gain compared to vehicle treatment. Daily average food intake was not altered by ulotaront (C), while water intake was dose-dependently increased, approaching significance at the highest dose (D). Ulotaront dose-dependently reduced elevated fasting glucose and insulin levels and improved insulin resistance determined by HOMA-IR. Lir = Liraglutide (0.2 mg/kg, sc, qd). Data are means ± or + SEM. Two-way repeated measures ANOVA (A) or one-way ANOVA (B–G) followed by Dunnett's post-hoc test; ∗p < 0.05, ∗∗p < 0.01, ∗∗∗p < 0.001 vs vehicle (Veh); N = 12/group.

Given the consistent improvement in body weight and metabolic parameters seen in the above studies, we further examined ulotaront in the mouse diet-induced obesity (DIO) model. In contrast to the rat HFD model described earlier, DIO mice are weight-stable and exhibit increased adiposity, insulin resistance and hyperglycemia following long term access to HFD. Daily oral administration of ulotaront for 35 days dose-dependently and significantly reduced body weight gain (Figure 5A) and overall percent body weight change (Figure 5B) at 3 and 10 mg/kg (−5% and −7% difference from vehicle respectively). In contrast to the previous results in rats, this reduction in body weight was associated with a dose-dependent trend to increase average daily water intake, while prominent effects were not seen on food intake when assessed over the entire study duration (Figure 5C–D). However, a significant decrease in food intake was observed during the first 2 days of dosing for the 3 and 10 mg/kg ulotaront groups (Supplementary Figure 4A). On day 15, ulotaront treatment significantly reduced fasting plasma glucose (1, 3 and 10 mg/kg) and insulin (10 mg/kg) levels (Figure 5E–F), suggesting improved insulin sensitivity. This was further supported by a dose-dependent reduction in HOMA-IR (Figure 5G). In line with reported findings [[49], [50], [51]], the GLP-1R agonist liraglutide produced significant and robust reductions in body weight (−17% difference vs vehicle), food intake, fasting plasma glucose and insulin levels. These results show that chronic treatment with ulotaront lowers body weight and improves glycemic control in the mouse DIO model.

### 3.7 Ulotaront and RO5263397 modulate neuronal activity in circuits implicated in energy balance and food intake

**Figure 6:**
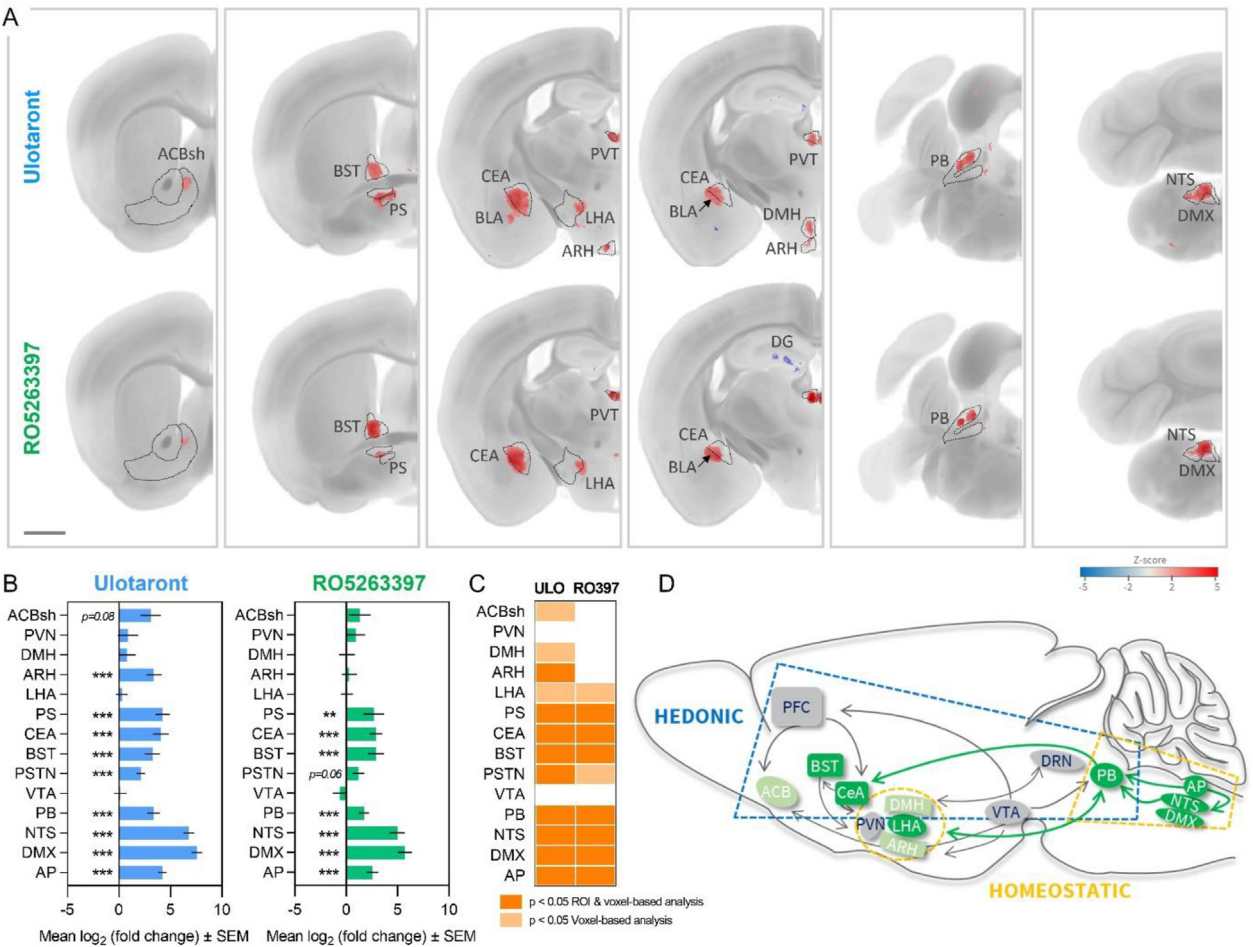
**Ulotaront and RO5263397 modulate activity in neurocircuits governing energy balance and feeding.** Whole-brain c-fos expression profiles following single administration of ulotaront (3 mg/kg, ip) or RO5263397 (0.3 mg/kg, ip). (A) Voxel-level coronal maps of significant differences compared to vehicle (z-score > 1.95) shown from anterior to posterior. Red and blue indicate increased and decreased c-fos expression, respectively. (B) Fold-change (log2 scale, mean ± SEM) in c-fos positive cell counts in appetite-regulating brain regions compared to corresponding vehicle controls. Dunnett's test negative binomial generalized linear model with p-value adjustment for multiple comparisons using FDR <0.05 (∗∗p < 0.01, ∗∗∗p < 0.001), N = 8/group. Scale bar = 1 mm. (C) Summary of compound-induced c-fos induction changes across the 14 individual brain regions. (D) Schematic of key brain regions and neurocircuits implicated in homeostatic and hedonic regulation of food intake. Brain regions significantly regulated by ulotaront and RO5263397 vs ulotaront only are shown in dark and light green, respectively. Abbreviations: ACB, nucleus accumbens; ACBsh, nucleus accumbens shell; ARH, arcuate hypothalamic nucleus; AP, area postrema; BLA, basolateral amygdalar nucleus; BST, bed nucleus of the stria terminals; CeA, central amygdalar nucleus; DMH, dorsomedial nucleus of the hypothalamus; DMX, dorsal motor nucleus of the vagus nerve; DRN, dorsal raphe nucleus; LHA, lateral hypothalamic area; NTS, nucleus of the solitary tract; PB, parabrachial nucleus; PFC, prefrontal cortex; PS, parastriatal hypothalamic nucleus; PSTN, parasubthalamic nucleus; PVN, paraventricular hypothalamic nucleus; PVT paraventricular nucleus of the thalamus; SNc, substantia nigra pars compacta; VTA, ventral tegmental area.

**Supplementary Figure 5:**
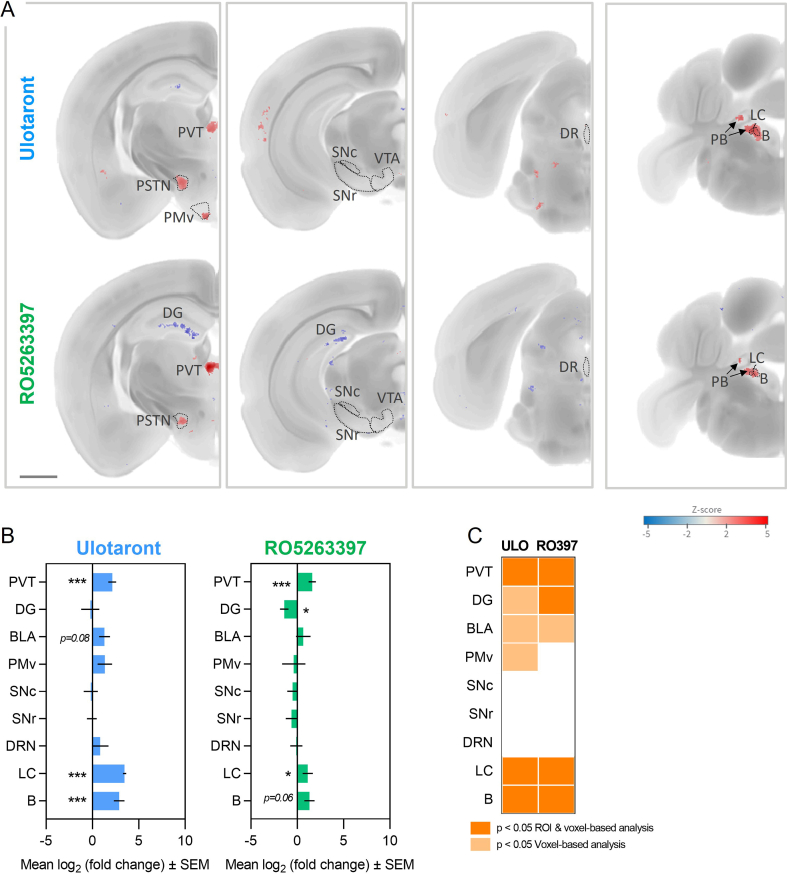
Effects of ulotaront and RO5263397 on c-fos expression in additional brain regions. Whole-brain c-fos expression profiles following single administration of ulotaront (3 mg/kg, ip) or RO5263397 (0.1 mg/kg, ip) in lean male mice. (A) Voxel-level coronal maps of significant differences compared to vehicle (z-score > 1.95) shown from anterior to posterior. Red and blue indicate increased and decreased c-fos expression, respectively. (B) Fold-change (log2 scale, mean ± SEM) in c-fos positive cells counts in regions of interest compared to corresponding vehicle controls. Dunnett's test negative binomial generalized linear model with p-value adjustment for multiple comparisons using FDR <0.05 (∗p < 0.05, ∗∗∗p < 0.001), N = 8/group. Scale bar = 1 mm. (C) Summary of compound-induced c-fos induction changes in 8 selected brain regions. B, Barrington's nucelus; D, dentate gyrus; DRN, dorsal raphe nucelus; BLA, basolateral amygdalar nucleus; LC, locus ceruleus; PB, parabrachial nucleus; PMv, ventral premammillary nucleus; PSTN: parasubthalamic nucleus; PVT, paraventricular nucleus of the thalamus; SNc, substantia nigra compacta; SNr, substantia nigra reticularis.

It is well recognized that the CNS plays a critical role in regulating energy balance, feeding behavior and body weight homeostasis [52,53]. The majority of centrally acting anti-obesity drugs exert appetite suppressing effects or affect food reward sensitivity [54,55]. Given the neuromodulatory actions of TAAR1, we also evaluated ulotaront (3 mg/kg) and RO5263397 (0.3 mg/kg) for effects on whole-brain c-fos protein expression after acute intraperitoneal administration. The expression of the immediate early gene c-fos serves as a marker of neural activity and was analyzed at single-cell resolution using c-fos immunohistochemistry and automated quantitative 3D imaging in male C57BL/6J mice. Since c-fos signals can be spatially restricted to parts of any given brain area, or span multiple areas, we conducted region-, and voxel-based statistical analysis. The region-based analysis comprised 839 atlas-defined mouse brain areas. Ulotaront and RO5263397 induced a strikingly similar c-fos profile, while some notable differences were also observed (Figure 6A–C). Both compounds modulated neural activity in key brain areas involved in energy homeostasis and hedonic feeding [52,56,57] (Figure 6D and Supplementary Figure 5). These included hypothalamic feeding centers, the extended amygdala and several appetite-regulating brainstem nuclei. The latter included the parabrachial nucleus (PB), nucleus of the solitary tract (NTS), dorsal motor nucleus of the vagus nerve (DMX) and area postrema (AP). Ulotaront and RO5263397 also robustly activated the extended amygdala complex including the central amygdala nucleus (CEA) and bed nucleus of the stria terminalis (BST). In addition, increased c-fos expression in response to ulotaront was detected in the dorsomedial (DMH) and arcuate (ARH) hypothalamic nuclei, parasubthalamic nucleus (PSTN) and the lateral hypothalamic area (LHA). More subtle hypothalamic signals, restricted to the PSTN and LHA, were seen with RO5263397. The differences in hypothalamic profiles do not likely reflect dose-specific effects of the two compounds given that the signals elicited in the brainstem and amygdala were of similar magnitude. Increased c-fos expression in response to ulotaront, and to a lesser extent RO5263397, was also observed within a restricted area of the nucleus accumbens shell (ACBsh), a major projection site of dopaminergic neurons. In addition, both compounds upregulated c-fos expression in the paraventricular nucleus of the thalamus (PVT) and locus ceruleus (LC) (Supplementary Figure 5). Statistically significant decreases in c-fos expression were rare, highly scattered and primarily observed at the voxel level. Of these, the most notable were seen in response to RO5263397 in the dentate gyrus (DG) (Figure 6A and Supplementary Figure 5). However, since baseline c-fos levels are generally low in naïve mice, treatment-induced decreases in c-fos expression are difficult to interpret and may reflect attenuation of stress-induced neuronal activation elicited by the test article injection. No prominent effects of ulotaront or RO5263397 were seen in midbrain dopaminergic (ventral tegmental area and substantia nigra) or serotonergic nuclei (dorsal raphe nucleus) (Supplementary Figure 5).

## 4 Discussion

Metabolic Syndrome, characterized by central obesity, dyslipidemia, hypertension and hyperglycemia, is highly prevalent in patients with schizophrenia and can be induced or exacerbated by the current class of antipsychotic drugs (APDs) [23,26,30]. The prevalence is as high as 69% in those with chronic illness and estimated to be 3–5 times higher than in the general population [25]. This represents a significant problem as it increases the risk of cardiovascular disease which likely contributes to the decreased life expectancy of schizophrenia patients [25,58]. However, marked differences exist between APDs in terms of metabolic side-effects with more recent work providing evidence that antipsychotic-mediated improvements in psychopathology are associated with metabolic disturbance [23]. Notably, the authors do not suggest that metabolic disturbance is a requirement for efficacy, but rather highlight that the most efficacious APDs tend to have the broadest polypharmacology, and that metabolic effects might be due to off-target actions. This emphasizes the need for a pharmacologically distinct class of compounds in the treatment of schizophrenia.

TAAR1 agonists, including the Phase 3 clinical development candidate ulotaront, have recently emerged as a potential new treatment approach in schizophrenia and other psychiatric disorders including depression, anxiety and substance abuse [1,59,60]. Intriguingly, recent preclinical evidence has also identified TAAR1 as a novel regulator of metabolic control and a potential target for obesity and type 2 diabetes [16,17,61,62]. Here we expand on these findings to show that TAAR1 agonists as a class reduce body weight and improve glycemic control by exerting peripheral effects on glucose homeostasis and gastric emptying, as well as directly modulating homeostatic and hedonic neurocircuits regulating energy balance and feeding. We primarily focused on the characterization of ulotaront given its advanced clinical development stage [1,5]. Ulotaront was discovered through a unique, target-agnostic approach optimized to identify drug candidates that lack D_2_ and 5-HT_2A_ receptor antagonism (hallmarks of the antipsychotic class) while demonstrating a phenotypic antipsychotic-like profile *in vivo* [6]. Subsequently, its primary receptor mechanism was identified to be TAAR1 agonism with additional 5-HT_1A_ receptor agonism [6,9,[63], [64], [65]]. Notably, the metabolic profiles of ulotaront and the selective TAAR1 agonists, RO5166017 and/or RO5263397, were strikingly similar including the effects on body weight, glucose, insulin, gastric emptying and c-fos expression. Although 5-HT_1A_ receptors have been implicated in energy balance, partial and full agonists have generally been shown to promote food intake and hyperglycemia in rodents while antagonists produced the opposite effect [[66], [67], [68], [69]]. In addition, the 5-HT_1A_ agonist, and antianxiety agent, buspirone has not been associated with clinically meaningful or consistent effects on body weight and metabolic parameters [70,71]. Together this strongly suggests that the beneficial effects of ulotaront on metabolic regulation are TAAR1-mediated.

Single administration of ulotaront and RO5166017 improved oral glucose tolerance in lean and/or diabetic *db/db* mice in an insulin-independent manner. The reductions in glucose excursion were likely triggered by a delay in gastric emptying, which was observed with all three TAAR1 agonists. Similar effects were previously shown for RO5166017, implicating gastric emptying as the main mechanism by which glucose excursion is reduced under these experimental (acute dosing) conditions [16]. However, when the stomach is bypassed by an iv glucose challenge, RO5166017 was reported to increase insulin secretion, which is not consistent with our results. Although the authors propose that TAAR1 agonists exert insulin secretagogue-like effects *in vivo*, the robustness of the effect is unclear as only one timepoint (i.e., 10 min) was assessed following single, subcutaneous administration of RO5166017 at a high dose (3 mg/kg) [16]. In addition, no significant decrease of glucose was noted during the ivGTT, which is consistent with our findings. However, both RO5166017 and RO5256390 were previously shown to increase glucose-stimulated insulin secretion in rat pancreatic β-cells and/or human islets [16,62]. This has led to the hypothesis that TAAR1-agonists exert incretin-like effects [16]. Incretin hormones, including GLP-1 and GIP, improve glycemia via their ability to enhance insulin secretion and by inhibiting gastric emptying to slow glucose entry to the general circulation [72,73]. Further work with all three agents is needed to fully elucidate TAAR1-mediated insulin secretagogue effects *in vitro* and *in vivo*.

Our data also show that prolonged (15 days) treatment with ulotaront improves insulin sensitivity in DIO mice. Elevated fasting glucose and insulin levels, as well as the HOMA-IR, were dose-dependently decreased by ulotaront. In addition, a significant and dose-dependent reduction in body weight was also observed. The magnitude of body weight loss compared to vehicle (−7%) and the improvement in glycemic control following ulotaront treatment are consistent with those described for RO5166017 in DIO mice [16]. Weight loss effects observed in DIO models have generally translated to humans although the maximal efficacy is historically greater in rodents [73]. The greatest effects reported in rodents and humans are with incretin-based therapies including the GLP-1R agonist semaglutide and the dual GIP/GLP-1R agonist tirzepatide [73]. Although the magnitude of weight loss achieved with ulotaront (−7%) was notably smaller compared to liraglutide (−17%) in this study, the effect was similar to those reported for other weight-lowering drugs in rodent DIO models including orlistat (gastrointestinal lipase inhibitor), lorcaserin (5-HT_2C_ receptor agonist) and naltrexone/bupropion (opioid receptor antagonist and dopamine/norepinephrine reuptake inhibitor) [73]. Ulotaront-mediated decreases in body weight and body weight gain were also seen in rats fed a HFD, as well as in two rodent models of iatrogenic weight gain. Chronic ulotaront administration (21 days) normalized corticosterone-induced body weight-gain in mice. In addition, we show that switching from olanzapine to ulotaront or RO5263397 reverses the olanzapine-induced body weight-gain in rats. Importantly, these effects were more pronounced compared to those observed in controls that just discontinued olanzapine treatment. These studies were designed to model a more clinically relevant situation where treatment switches are common. RO5263397 and ulotaront have also been reported to attenuate olanzapine-induced body weight gain in rodents when co-administered for 14 and 34 days, respectively [17,21]. As suggested by Revel and colleagues, TAAR1 agonists represent the first pharmacological class that is associated with antipsychotic-like activity while demonstrating protection from atypical antipsychotic-induced body-weight gain.

The body weight lowering effects of TAAR1 agonists are likely associated with decreased food intake and the slowing of gastric emptying, as is seen for GLP-1R agonists. Ulotaront and RO5263397 reduced food consumption in the rat HFD model without notably affecting water intake. Potentially, as a consequence of body weight reduction, liver triglyceride levels were improved with ulotaront consistent with reports from earlier RO5166017 studies [16]. Interestingly, the effect of ulotaront on food intake was not as pronounced in DIO mice and only noted during the first few days of dosing. In contrast, water intake was dose-dependently increased throughout the study duration, approaching statistical significance. These initial observations suggest that TAAR1 agonists may affect energy expenditure however this possibility requires further investigation.

In light of preclinical findings, it is worth noting that treatment with ulotaront was not associated with clinically meaningful changes in body weight or metabolic parameters (i.e. glucose, triglycerides, cholesterol or HbA1c) in a 4 week randomized, placebo-controlled clinical trial in schizophrenia patients or the subsequent 26-week open label extension [5,7]. While this phase 2 study supports a differentiated metabolic-risk profile for ulotaront compared to APDs [8,23,26], the majority of subjects were not obese (i.e. mean BMI at baseline = 25) or hyperglycemic which likely precluded the ability to detect treatment-induced weight loss or metabolic improvements [5,7]. In addition, the treatment regimen and trial duration required to demonstrate potential effects of ulotaront on metabolic endpoints are currently unknown. Clinical studies in patients with metabolic dysfunction are therefore needed to further explore the potential benefits of TAAR1 agonists, including ulotaront. In this regard, dedicated phase I studies in schizophrenia patients with metabolic syndrome are currently ongoing to evaluate the effects of ulotaront on gastric emptying, glycemic control, and weight-associated parameters (NCT05402111, NCT05463770, NCT05542264).

In addition to producing direct peripheral, paracrine or endocrine effects in the gastrointestinal tract, we also assessed whether TAAR1 activation modulates key neuronal circuits implicated in the regulation of energy balance and food intake. Using c-fos protein expression as an indirect marker of neuronal activity, we mapped the whole-brain activation signatures of ulotaront and RO5263397 in mice. Largely overlapping expression profiles were seen for both compounds further suggesting TAAR1-mediated effects. Increased c-fos levels were detected in several interconnected nuclei and neurocircuits involved in the regulation of homeostatic feeding and reward sensitivity. These included discrete hypothalamic nuclei, central amygdala, BST, PB and the brainstem dorsal vagal complex (DVC) containing the NTS, AP and DMX. Modulation of vagal neurocircuitry, including the complex interplay between the DVC, enteric nervous system, hypothalamus and limbic forebrain, is heavily implicated in the regulation of gastric motility and satiety [74]. Activation of the DVC, particularly the NTS, is associated with prominent satiation signals, which are transmitted downstream to the PB, CEA and hypothalamus to promote meal termination [52,53,75]. Notably, increased DVC activity and DVC-limbic forebrain connectivity, has been proposed as a potential mechanism underlying the anorectic effects and nausea responses of GLP-1R agonists [34,40,[76], [77], [78], [79], [80]]. The extent to which TAAR1 agonists are associated with nausea and/or emesis is subject to further research. Recent work in rats suggests that RO5166017 and RO5263397 can induce conditioned taste aversion [81]. In phase 1 and 2 clinical studies, gastrointestinal-related adverse events, including nausea, were observed in some subjects receiving ulotaront, but these were generally mild or moderate in severity [5,7,8,82,83].

Engagement of hypothalamic mechanisms has been repeatedly implicated in the weight-lowering properties of several drugs. Key hypothalamic nuclei engaged in feeding control and energy expenditure were modulated by ulotaront including the ARH, DMH and LHA [54,55]. These brain regions are tightly interconnected and receive and/or transmit multiple peripheral inputs enabling nutrient sensing and feeding regulation. Notably, only discrete subareas of the DMH and LHA were activated in response to ulotaront which may suggest regulation of specific neuronal subpopulations. In addition, upregulation of c-fos was observed in the PS and PSTN. Activation of the PSTN has been linked to the suppression of hedonic feeding behavior, mediated via connectivity to the CEA and the insular cortex [84,85]. The effects of RO5263397 within the hypothalamus were restricted to the PS, LHA and PSTN, representing the most prominent differentiation between the two compounds. This may relate to the different pharmacological profiles of (i.e., selective vs non-selective TAAR1 agonist) and/or the doses tested. In addition, ulotaront and RO5263397 are full and partial TAAR1 agonists, respectively. Although differences between TAAR1 full and partial agonists have been described in slice electrophysiology studies [17,86], the reported behavioral profiles *in vivo* are generally consistent [1], in line with our current results. Ulotaront, and to a lesser extent RO5263397, also increased c-fos expression within the ACBshell, which receives prominent dopaminergic input from the VTA. The VTA-ACB mesolimbic dopamine pathway is critically involved in the incentive, reinforcing and motivational aspects of food intake. Prominent effects of TAAR1 agonists on the dopaminergic system have been reported, including decreased VTA neuronal firing and presynaptic dopamine synthesis capacity and release [1,12,17,37,87]. Modulation of dopaminergic circuits is likely associated with the antipsychotic-like effects of TAAR1 agonists as well as with the beneficial effects on compulsive, binge-like eating reported for RO5256390 in rats [20]. Ulotaront and RO5263397 also increased c-fos expression in the PVT, which represents a key thalamic relay station, connecting the limbic forebrain with hindbrain nuclei [88].

The c-fos expression profiles elicited by ulotaront and RO5263397 share a high degree of overlap with those previously reported for several weight-lowering compounds [34]. Most notably, this includes agents with diverse mechanisms such as the GLP-1R agonist semaglutide, bromocriptine (dopamine receptor agonist) and rimonabant (cannabinoid CB_1_ receptor antagonist). Lack of blood–brain barrier penetrability of semaglutide suggests that c-fos signals in the BST, CEA, PS and PB are secondary to direct effects in the circumventricular/paraventricular areas (i.e. NTS/DMX and hypothalamus) [51]. Both, ulotaront and RO5263397 readily cross the blood–brain barrier [6,17,89] suggesting direct modulation of c-fos expression. Future studies investigating time- and dose-response relationships on c-fos expression are warranted to further enable interpretation of TAAR1-mediated effects on whole-brain neuronal activity. Characterization of the specific cell-types modulated by ulotaront and RO5263397 is necessary to advance the mechanistic understanding of TAAR1-mediated metabolic regulation. In addition, other imaging approaches are needed to profile any inhibitory effects of TAAR1 agonist on neuronal activity given that baseline c-fos expression is generally too low to enable accurate detection of decreased activity.

In summary, we show that TAAR1 agonists as a class, including the clinical drug-candidate ulotaront, not only lack APD-induced metabolic liabilities but can reduce body weight and improve glycemic control in rodent models of diabetes, obesity, and/or iatrogenic weight gain. In vivo pharmacology and whole-brain c-fos imaging studies link the underlying mechanisms to TAAR1-mediated peripheral effects on glucose homeostasis and gastric emptying as well as direct modulation of homeostatic and hedonic neurocircuits regulating energy balance and feeding. The current preclinical evidence suggests that TAAR1 agonists may not only hold promise to improve schizophrenia symptoms, but potentially also comorbid metabolic dysfunction. If translated to humans, the beneficial metabolic effects of TAAR1 agonists may represent an improved risk–benefit profile compared to established antipsychotic drug classes.

## Funding

This work was supported by funding from Sumitomo Pharma America Inc. and Otsuka Pharmaceuticals Development & Commercialization, Inc.

## Author contributions

ND, LW, EHK, SCH and LJB conceived the project and designed the experiments. SPV performed the gastric emptying (phenol red), glucose tolerance and body weight studies in HFD rats and DIO mice. JHS and UR performed the gastric emptying (AAT assay) and/or c-fos imaging studies. CA and SW performed the mouse chronic corticosterone experiment. ND, LW, EHK, PGJ, SM, CS, SV, JHS, CA and LJB analyzed and/or interpreted the experimental data. ND, LJB and KSK supervised the research. ND wrote the manuscript with contributions from all authors. All the authors read and approved the final manuscript.

## Declaration of competing interest

ND, LW, PGJ, CS, SM, SCH, LJB and KSK are employees of Sumitomo Pharma America, Inc (formerly Sunovion Pharmaceuticals). EHK was an employee of Sunovion Pharmaceuticals at the time the studies were conducted. JHS and UR are employees of Gubra ApS. SPV is an employee of Sygnature Discovery. CA has received investigator-initiated research funding from Sumitomo Pharma America, Inc.

## Data Availability

Data will be made available on request.
